# Enhancing Aluminum Cutting Quality Through XGBoost-Assisted Optimization of Ultrafast Femtosecond Laser Processing

**DOI:** 10.3390/mi17070837

**Published:** 2026-07-14

**Authors:** Hyunbin Kang, Eunyeop Ji, Vassilia Zorba, Dongkyoung Lee, Minok Park

**Affiliations:** 1 Department of Mechanical and Automotive Engineering, Kongju National University, Cheonan 31080, Republic of Korea; hbee3726@gmail.com (H.K.); eunyeopji1215@gmail.com (E.J.); 2 Laser Technologies Group, Energy Technologies Area, Lawrence Berkeley National Laboratory, Berkeley, CA 94720, USA; vzorba@lbl.gov; 3 Department of Mechanical Engineering, University of California at Berkeley, Berkeley, CA 94720, USA

**Keywords:** femtosecond laser processing, eXtreme Gradient Boost machine learning, aluminum foil, debris quantification

## Abstract

The slitting of aluminum (Al) foil is a critical process in secondary battery manufacturing, where cut quality directly affects electrode uniformity and production yield. Although femtosecond (fs) laser processing has emerged as a promising approach for high-precision foil cutting, residual debris generated during material removal can degrade product quality and requires accurate process evaluation. In this study, a hybrid framework combining adaptive computer vision and eXtreme Gradient Boosting (XGBoost) was developed for automated debris quantification, quality classification, and process optimization of fs laser-processed Al foils. The image processing algorithm automatically detects debris boundaries from scanning electron microscopy images and extracts key geometrical descriptors, which are subsequently used as input features for XGBoost models. The developed framework successfully distinguished acceptable and defective processing conditions and accurately predicted residual debris sizes. Experimental validation under previously unseen processing conditions confirmed excellent agreement between predicted and measured debris sizes. By enabling automated and interpretable quality assessment, the proposed framework provides a scalable foundation for defect quantification and machine-learning-assisted process optimization in advanced laser manufacturing.

## 1. Introduction

In modern semiconductor and electronic device manufacturing, precision processing of thin metallic materials, particularly aluminum (Al), is increasingly required for a wide range of applications, such as the fabrication of current collectors for high capacity rechargeable batteries [1,2], microrobot [3], wettability [4], and the micropatterning and repair of thin film structures in microelectronic devices [5]. Although various fabrication techniques have been employed to process Al, its limited thickness and low thermal mass make it susceptible to thermal damage during material removal processes [6,7]. Consequently, minimizing thermal and morphological damage, including heat-affected zones (HAZs), burrs, and residual debris, is essential for ensuring product quality and maintaining the stability of subsequent manufacturing processes [8,9,10].

Recently, ultrashort femtosecond (fs) laser processing has attracted considerable attention as an effective approach for minimizing thermal damage during material processing [11,12,13,14]. Its ultrashort pulse duration enables energy deposition within a timescale on the order of hundreds of fs, thereby effectively suppressing heat diffusion into the surrounding material [15,16]. Therefore, fs laser processing enables high precision micromachining with minimal HAZ formation [12] and has facilitated advanced applications, including next-generation energy harvesting and storage systems [17,18], biomedical devices [19,20], machine learning (ML)-assisted manufacturing [21,22], and surface wettability control [23].

Despite these advantages, processing-induced damage cannot be completely eliminated even with fs laser processing [24,25]. In the case of Al, its high thermal conductivity (~237 W m^−1^ K^−1^ at room temperature) and low melting point (~933 K) can promote complex melting, material ejection, and re-solidification behaviors during laser ablation [26,27]. As a result, vaporized and molten material often redeposits or rapidly solidifies near the cut edge, forming micro-/nanoscale residues [26,28]. In electronic and packaging applications, these unwanted residues can act as contamination sources and may lead to electrical short circuits, bonding failures, and long-term reliability degradation [8]. Therefore, a quantitative method for evaluating residue formation is essential for process optimization and quality control [8,26,29].

Conventional residue evaluation primarily relies on manual measurements based on visual inspection of cross-sectional scanning electron microscopy (SEM) images [30]. However, such approaches are susceptible to subjective bias, often leading to inconsistent evaluation results [31,32]. Furthermore, accurately defining the baseline and residue boundaries is challenging because of complex surface morphologies [28] and indistinct interfaces [33,34]. In addition, analyzing a large number of specimens for process optimization requires substantial time and labor, limiting the practicality of conventional evaluation methods [31,32,34].

To overcome these limitations, ML based convolutional neural networks (CNNs) have recently been applied to process quality inspection tasks, owing to their ability to automatically extract complex image features [35,36]. However, CNNs typically require large amounts of labeled data for reliable training, making them prone to overfitting in data-limited laboratory environments [37,38]. Furthermore, their performance could be sensitive to variations in SEM image quality and specimen morphology [39,40]. Moreover, their black box nature limits the interpretation of the geometrical features and defect mechanisms underlying classification results [41,42]. Therefore, there is a need for ML approaches that can accurately evaluate residue formation using limited datasets while maintaining interpretability.

Herein, we propose a hybrid framework that combines an adaptive computer vision algorithm with an eXtreme Gradient Boosting (XGBoost) model for automated debris quantification and quality classification of fs laser-processed Al foils. The proposed image processing algorithm automatically detects debris boundaries from SEM images and extracts key geometrical descriptors of debris morphology. These descriptors are then used as input features for XGBoost to classify processing quality as either acceptable or defective. Unlike manual measurements, the proposed method enables objective and automated evaluation, while the interpretable nature of XGBoost provides insight into the geometrical factors governing residual debris formation. The proposed framework offers a foundation for data efficient and interpretable quality inspection and may facilitate the broader adoption of ML-assisted process optimization in advanced manufacturing applications.

## 2. Materials and Methods

### Ultrafast Fs Laser Processing of Al Foils

**Figure 1:**
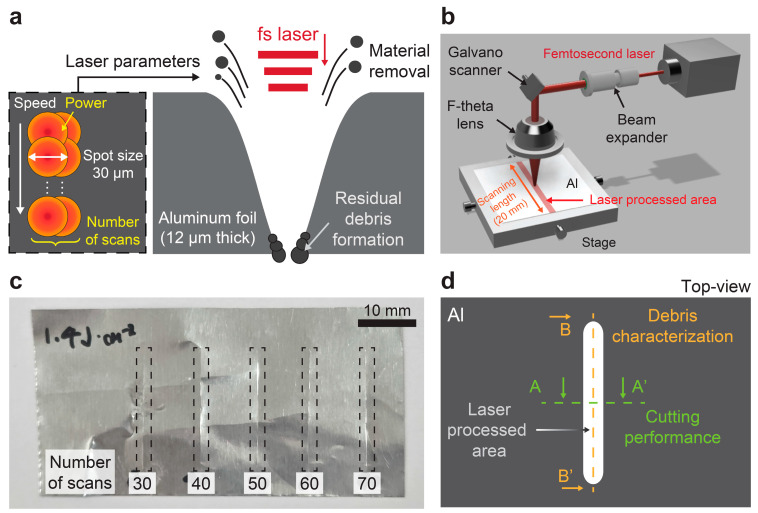
Experimental setup and processing concept for fs laser cutting of Al foils. (**a**) Schematic illustration of the laser processing parameters, including laser fluence and number of scans, which govern cutting performance and residual debris formation. The laser spot diameter was approximately 30 µm. (**b**) Schematic of the fs laser processing system integrated with a galvano scanner. (**c**) Representative optical image of Al foils processed under different laser conditions. (**d**) Characterization directions used for subsequent SEM analysis of cutting performance and residual debris morphology. The A–A’ direction was used to evaluate cutting performance, while the B–B’ direction was used to characterize residual debris morphology.

Thin Al foils for battery cathode substrates with 12 µm in thickness (MTI Korea, Seoul, South Korea) were processed using the fs laser system to achieve high-precision cutting with minimal thermal damage, as illustrated in Figure 1a. Due to the non-thermal ablation characteristics of the fs laser and the high thermal conductivity of Al, the 12 µm thin foil exhibited no thermally induced warping or deformation during repeated scanning.

Compared with conventional laser processing techniques, such as nanosecond lasers, the ultrashort pulse duration (200 fs) enables energy deposition on a shorter timescale, thereby limiting heat transfer to the surrounding material and minimizing undesirable thermal effects [13]. As a result, fs laser processing provides an effective approach for cutting metallic foils while maintaining high dimensional accuracy. As shown in Figure 1a, the cutting performance and residual debris formation are influenced by incident laser parameters, including laser fluence, scanning speed, and number of scans.

A schematic overview of the experimental setup is presented in Figure 1b. The fs laser source (Carbide, Light Conversion, Vlinius, Lithuania) operated at a wavelength of 1030 nm with a pulse duration of 200 fs and a repetition rate of 1 MHz. Prior to reaching the sample, the laser beam was expanded using a beam expander (3×) and subsequently directed to a galvano-scanning system (SG8220, Sino-Galvo, Zhenjiang, Jiangsu, China) equipped with a f-theta lens, resulting in a focused spot diameter of approximately 30 μm on the sample surface. The scanning length was fixed at 20 mm to ensure continuous cutting along the entire processing path.

The cutting behavior was controlled by varying the incident laser power, corresponding laser fluence (J cm^−2^), and the number of repeated scans along the same cutting trajectory. By systematically changing these parameters, the influence of accumulated laser energy on foil penetration and residual debris formation was investigated. Detailed laser processing conditions are summarized in Table 1. All experiments were conducted without assist gas at a constant scanning speed of 6 m s^−1^.

**Figure 2:**
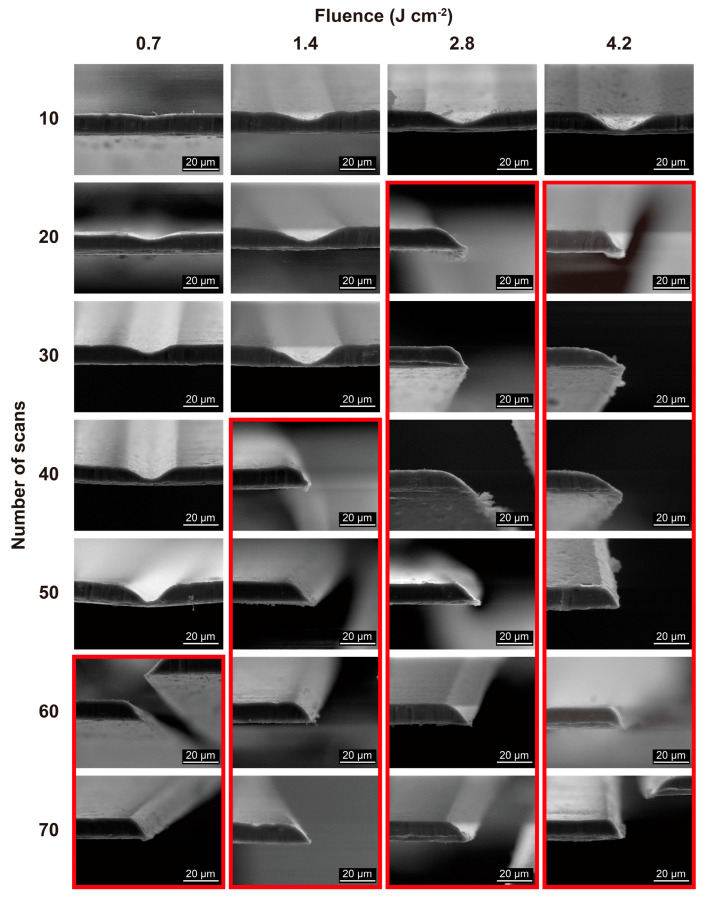
Cutting performance of fs laser-processed Al foils under different laser processing conditions. The Al foils were characterized using SEM as a function of laser fluence and number of scans. Images highlighted with red rectangles indicate processing conditions under which complete foil cutting was achieved. The white scale bar is 20 µm.

**Figure 3:**
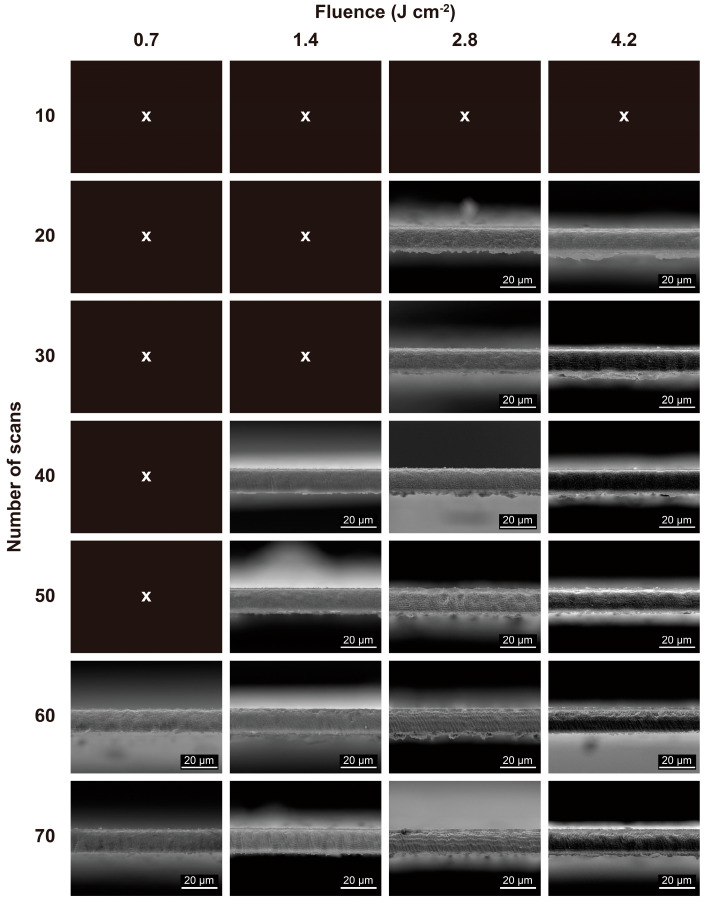
Cross-sectional SEM images of fs laser-processed Al foils obtained under different laser fluence and scan-number conditions. Processing conditions that did not result in complete foil cutting are marked with an “X”. The white scale bar is 20 µm.

Figure 1c presents a representative optical image of the processed Al foil at a laser fluence of 1.4 J cm^−2^, showing the influence of the number of scans on cutting quality. Figure 1d schematically shows the characterization directions used for subsequent SEM analysis. The A–A′ direction was employed to evaluate cutting performance and foil penetration (Figure 2), whereas the B–B′ direction was used to characterize residual debris morphology, including the average debris height and its corresponding standard deviation (Figure 3).

## 3. Results

### 3.1. Cutting Performance of Fs Laser-Processed Al Foils

After fabrication, the laser-processed Al foils were characterized using SEM to investigate the effects of laser fluence and number of scans on cutting performance, as shown in Figure 2. Red rectangles indicate the processing conditions under which complete foil cutting was achieved. Overall, the cutting behavior was strongly influenced by both laser fluence and the number of scans, highlighting the importance of accumulated laser energy during material removal.

Specifically, at lower laser fluences and scan numbers, the Al foils could not be fully penetrated. For example, when only 10 scans were applied, complete cutting was not achieved across the entire fluence range investigated (0.7–4.2 J cm^−2^). This result indicates that the accumulated laser energy was insufficient to remove the full foil thickness through laser ablation. As the number of scans increased, the ablation depth gradually increased, leading to improved cutting performance. For instance, under a fluence of 0.7 J cm^−2^, progressively deeper ablation features were observed as the number of scans increased from 10 to 70.

A similar trend was observed with increasing laser fluence. Higher fluence conditions enhanced material removal by delivering greater energy per pulse, thereby reducing the number of scans required for complete foil penetration. For example, successful cutting was achieved at a fluence of 2.8 J cm^−2^ with only 20 scans, whereas a higher number of scans (70 scans) was required at a lower fluence of 0.7 J cm^−2^. These results indicate that both laser fluence and scan number play critical roles in determining the accumulated energy delivered to the material and, consequently, the cutting performance.

Although higher fluence and scan numbers facilitated complete foil cutting, SEM observations revealed the presence of residual debris along the cut edges. These residues are attributed to the redeposition and rapid re-solidification of molten and vaporized material generated during laser ablation. Furthermore, noticeable variations in debris morphology and accumulation were observed among different processing conditions, suggesting that cutting quality cannot be evaluated solely based on penetration performance. Therefore, a quantitative and objective assessment of debris formation is necessary to identify optimal processing conditions and ensure high-quality laser cutting of thin Al foils.

### 3.2. Residual Debris Characterization

**Figure 4:**
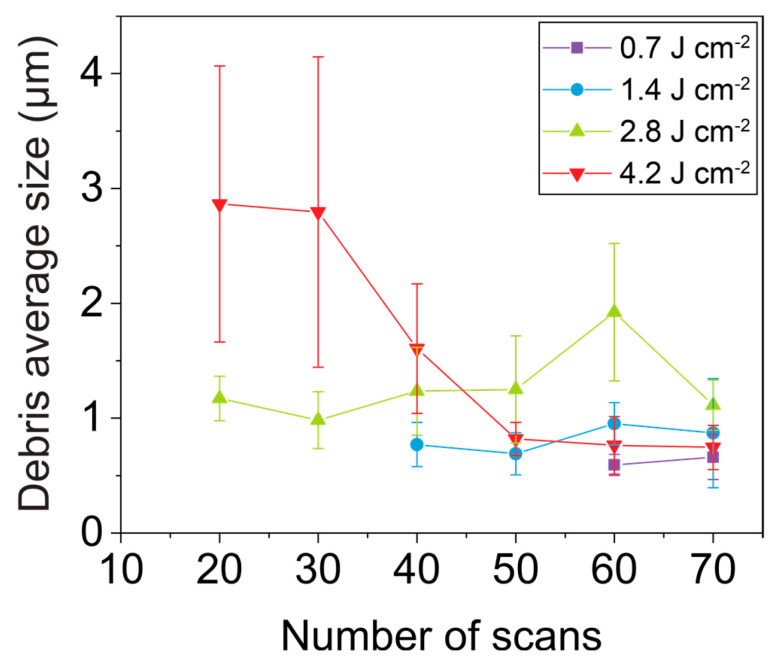
Quantitative characterization of residual debris morphology. The average debris height and its standard deviation were determined from the cross-sectional SEM images shown in Figure 3 for different laser processing conditions.

Figure 3 presents cross-sectional SEM images of fs laser-processed Al foils acquired to investigate residual debris formation under different processing conditions. To quantitatively evaluate these variations, a systematic analysis of debris size and morphology was subsequently performed, as presented in Figure 4. The amount and morphology of the residual debris varied with laser fluence and number of scans, indicating that debris formation is strongly influenced by the processing parameters.

Particularly, at lower laser fluences of 0.7 and 1.4 J cm^−2^, relatively clean cross-sectional surfaces were observed after successful foil cutting, with debris sizes typically below 0.8 µm. Only a small amount of residual debris was detected near the cut edges, indicating that material removal occurred in a relatively controlled manner under these conditions. The cut boundaries also appeared more uniform, suggesting limited redeposition of molten or vaporized material during the ablation process. Such behavior is advantageous for precision manufacturing applications, where excessive debris can adversely affect dimensional accuracy and subsequent assembly processes.

In contrast, higher laser fluence conditions (>2.8 J cm^−2^) resulted in noticeably greater debris accumulation along the cut edges, with debris sizes frequently exceeding 1.0 µm. As the incident fluence increased, a larger amount of material was removed per pulse, generating a greater volume of ejected molten and vaporized material. A portion of this material subsequently redeposited and rapidly resolidified on the surrounding surfaces, leading to the formation of irregular microscale debris structures.

Furthermore, the size of the residual debris tended to decrease with increasing number of scans, reaching values below 1.0 µm for conditions involving 70 scans. Repeated laser irradiation along the same cutting path continuously supplied energy to the processed region, promoting not only further material removal from the foil but also re-ablation of debris accumulated near the cut edge. Consequently, part of the deposited material was removed during subsequent scans, resulting in smaller and less pronounced debris structures.

Overall, these observations demonstrate that debris formation is governed by a complex interplay between laser fluence and scan number. While sufficient energy input is required to achieve complete foil penetration, excessively high fluence conditions tend to promote debris accumulation and deteriorate edge quality. Therefore, from a quality control perspective, higher fluence is not necessarily advantageous, and an appropriate balance between laser fluence and scan number is required to achieve high-quality cutting with minimal residual debris.

### 3.3. Architecture and Training of the XGBoost Framework

For structured, low dimensional experimental datasets, XGBoost is well suited to capturing complex nonlinear relationships while providing quantitative feature importance for model interpretation [43,44]. In contrast, manual measurements of debris morphology from cross sectional SEM images (Figure 3) are time consuming, labor intensive, and susceptible to operator dependent variability, as they rely on selected local measurements rather than the complete debris profile. To overcome these limitations, an adaptive computer-vision algorithm was developed to automatically extract debris size information from SEM images of fs laser processed Al foils.

**Figure 5:**
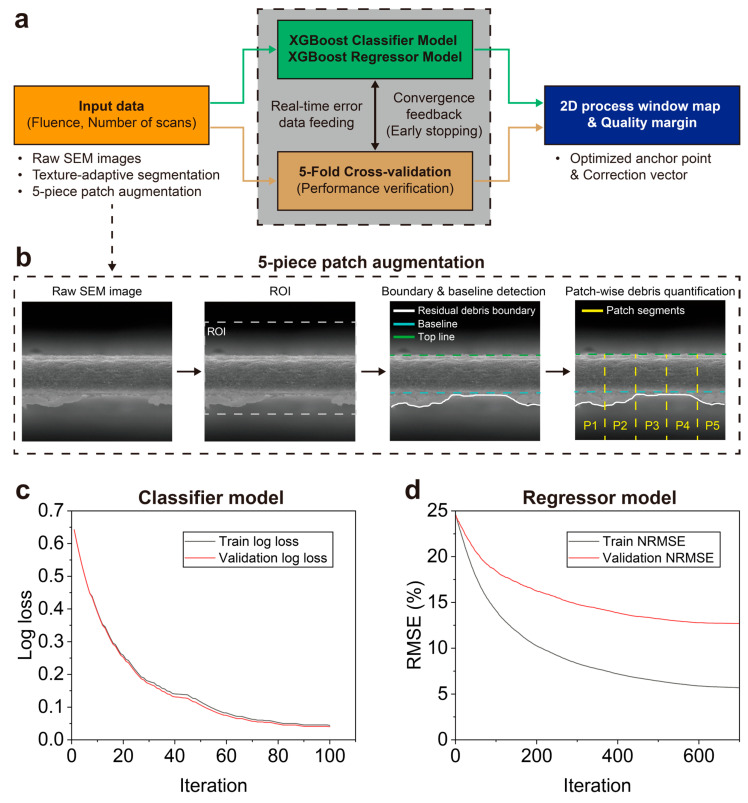
Architecture and training of the XGBoost-based optimization framework. (**a**) Schematic workflow of the proposed framework, mapping the processing parameters (laser fluence and number of scans) to the residual debris size through a dual XGBoost classifier–regressor architecture, with 5-fold cross-validation and early stopping for performance verification and convergence feedback. Arrows indicate the training process. (**b**) Image-processing and five-piece patch-augmentation pipeline: raw SEM image, ROI extraction, debris boundary and baseline detection via texture-adaptive segmentation, and patch-wise debris quantification over five segments (P1–P5). (**c**) Training and validation log loss curves for the XGBoost classifier, demonstrating the iterative minimization of classification error. (**d**) Training and validation RMSE curves for the XGBoost regressor, indicating stable model convergence as the number of training iterations increases.

Figure 5a illustrates the overall framework linking laser processing parameters to geometric quality metrics through a dual-model architecture. Laser fluence and number of scans were used as the primary process inputs, while residual debris size was selected as the target quality descriptor. Here, real time error feedback refers to iterative model-level updates during training and validation rather than in situ closed-loop control of the laser process.

To accurately quantify debris morphology, a multi-step image processing pipeline was implemented, as schematically shown in Figure 5b. The algorithm first identifies the region of interest (ROI) containing the processed cross-section and detects the foil surface profile. The outer debris boundary is subsequently extracted based on local contrast and texture variations, and a valley baseline is defined from the recessed regions of the detected profile. To augment the limited experimental dataset, the calibrated debris profile was divided into five equal-width spatial patches. The average debris size was then independently evaluated for each patch, increasing the number of training samples by a factor of five and generating a statistically enriched dataset for ML analysis.

The extracted patch-wise features were subsequently used to train two XGBoost models: a classifier for quality discrimination and a regressor for process parameter correction. To mitigate data imbalance and penalize poor processing conditions, a dynamic sample weighting strategy was incorporated into the regression model. The classifier employed a maximum tree depth of 3 with 100 boosting rounds and a learning rate of 0.05, whereas the regressor used a depth of 2 with 700 boosting rounds and a learning rate of 0.02. To assess model robustness under limited data availability, an adaptive K-fold cross-validation strategy with up to five folds was applied.

For process optimization, the regressor identified physically meaningful target anchor points by minimizing laser fluence and scan count while satisfying the quality constraint of debris size below 1.0 μm. Rather than predicting absolute process parameters, the model was trained to estimate the correction vectors (Δfluence and Δscan) required to reach the optimized anchor point. These target variables were normalized using min–max scaling prior to training.

The performance of the trained models is summarized in Figure 5c,d. The classifier exhibited rapid convergence, with both training and validation log loss values reaching approximately 0.04 within 100 boosting rounds. Similarly, the regressor achieved normalized root-mean-square errors (NRMSEs) of approximately 5.7% for training and 12.7% for validation after 700 rounds. Although a moderate generalization gap remained, the validation error reached a stable plateau without divergence, indicating that the early-stopping strategy effectively suppressed overfitting. The resulting framework enables both quality classification and parameter-correction prediction within the laser fluence–scan-number design space.

### 3.4. Performance Validation of the XGBoost-Based Optimization Framework

**Figure 6:**
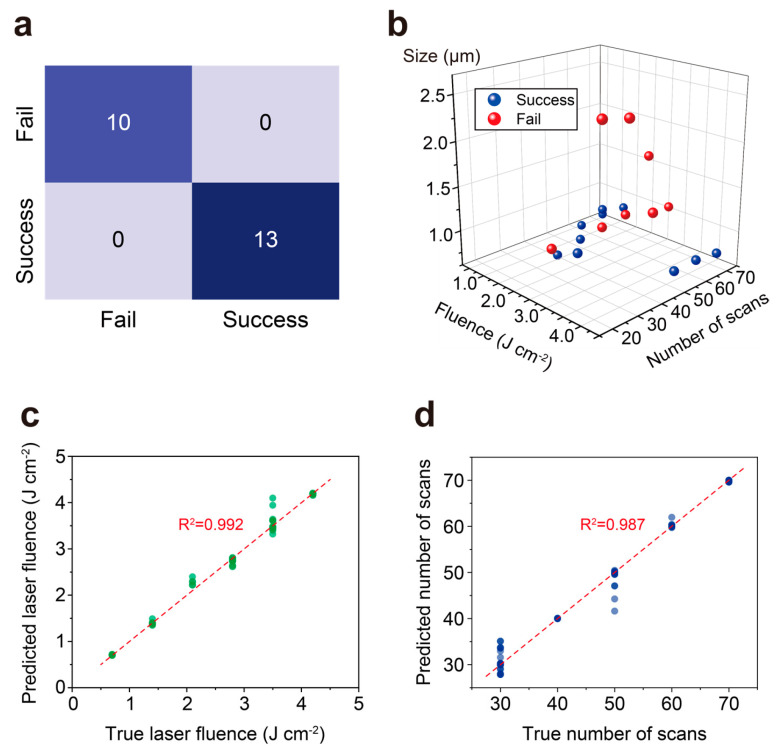
Performance validation of the XGBoost-based quality classification and optimization framework. (**a**) Confusion matrix demonstrating the classification performance for distinguishing acceptable and defective processing conditions. (**b**) Three-dimensional distribution of the measured debris height as a function of laser fluence and number of scans. (**c**) Parity plot comparing the target and predicted laser-fluence correction values generated by the XGBoost regressor (R^2^ = 0.992). (**d**) Parity plot comparing the target and predicted scan-number correction values generated by the XGBoost regressor (R^2^ = 0.987).

To evaluate the proposed framework, both the XGBoost classifier and regressor were tested using an independent dataset. As shown in Figure 6a, the classifier successfully distinguished acceptable and defective processing conditions, achieving perfect classification performance on the test set. To ensure a robust and unbiased assessment, the dataset was partitioned using a stratified sampling strategy to preserve class balance, and model reliability was further verified through fivefold cross validation.

The classification results are closely related to the process quality map shown in Figure 6b. Considering that the thickness of the aluminum foil used in this study is 12 µm, a debris size corresponding to 10% of the foil thickness (1.2 µm) could serve as a standard evaluation criterion. However, to ensure higher process reliability and product quality, a more conservative threshold of 1 µm was established as the criterion for separating acceptable and defective conditions. A clear separation between acceptable and defective processing conditions can be observed in the laser fluence–scan number space using a debris size threshold of 1 μm, which was selected based on the quantitative analysis presented in Figure 4. The resulting decision boundary demonstrates that the classifier effectively captured the relationship between laser processing parameters and residual debris formation.

Figure 6c,d present the regression results for laser fluence and scan number, respectively. In both cases, the predicted values exhibited excellent agreement with the corresponding experimental targets, yielding coefficients of determination (R^2^) of 0.992 for laser fluence and 0.987 for scan number. These results demonstrate that the proposed framework not only enables accurate quality classification but also provides reliable parameter-correction guidance for achieving high-quality fs laser cutting with minimal residual debris.

### 3.5. Experimental Validation of the XGBoost-Based Framework

**Figure 7:**
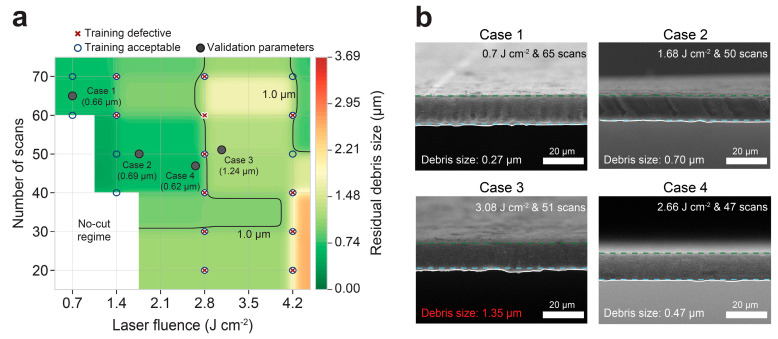
XGBoost-based prediction and validation of residual debris size. (**a**) Predicted residual debris-size distribution in the laser fluence—number of scans design space. (**b**) Comparison of model predictions with experimental measurements obtained from cross-sectional SEM images for four representative processing conditions. The green and blue dashed lines indicate the top and bottom surfaces of the Al foil, respectively. The white scale bar is 20 µm.

Finally, the predictive capability of the trained XGBoost model was evaluated by examining its ability to estimate residual debris sizes across unexplored laser processing conditions. Figure 7a presents the predicted debris size map in the laser fluence–scan number space. The model successfully interpolates between experimentally measured conditions, enabling the prediction of residual debris formation throughout the design space.

To experimentally validate the proposed framework and verify the predicted process boundary, four laser parameter sets were randomly selected from the prediction map: Case 1 (0.66 µm for 0.7 J cm^−2^ & 65 scans), Case 2 (0.69 µm for 1.68 J cm^−2^ & 50 scans), Case 3 (1.24 µm for 3.08 J cm^−2^ & 51 scans), and Case 4 (0.62 µm for 2.66 J cm^−2^ & 47 scans). These conditions were not included in the training dataset and were specifically chosen to assess the model’s predictive performance under previously unseen processing conditions.

As shown in Figure 7b, Cases 1, 2, and 4, which were predicted to lie within the acceptable process window, exhibited relatively clean cross-sectional morphologies. The measured debris sizes were 0.27 µm, 0.70 µm, and 0.47 µm, respectively, all below the quality threshold of 1.0 µm. These results confirm that suitable combinations of laser fluence and scan number can achieve high-quality cutting with minimal residual debris.

In contrast, Case 3, which was predicted to fall within the defective region, exhibited noticeable deterioration in edge quality. Irregular debris accumulation was observed along the cut edge, and the measured debris size reached 1.35 µm, exceeding the threshold criterion. Interestingly, although Case 3 was processed with both a higher laser fluence and a greater number of scans than Case 4, its cutting quality was inferior. This behavior suggests that excessive energy input promotes melting and redeposition of ablated material, resulting in increased residual debris despite repeated laser irradiation.

Importantly, the experimental observations showed good agreement with the model predictions. The absolute prediction errors were 0.39 µm, 0.01 µm, 0.11 µm, and 0.15 µm for Cases 1–4, respectively. Both acceptable and defective processing conditions were correctly identified, and the predicted process boundary was experimentally validated. These results demonstrate that the proposed framework can reliably predict residual debris formation and serve as a practical tool for process-window identification and parameter optimization in fs laser cutting of thin Al foils.

## 4. Conclusions

In this study, a hybrid framework combining adaptive computer vision and XGBoost was developed for automated residual debris quantification, quality classification, and process optimization of fs laser-processed thin Al foils. The proposed image processing algorithm successfully detected debris boundaries from SEM images and extracted key geometrical descriptors of debris morphology, enabling automated quality evaluation without manual measurements. The extracted descriptors were subsequently used as input features for XGBoost-based classification and regression models, which successfully distinguished acceptable and defective processing conditions and predicted parameter corrections for quality improvement. Experimental validation using previously unseen processing conditions showed good agreement between the predicted and measured debris sizes, confirming the reliability of the proposed framework. Compared with conventional manual inspection and data-intensive CNN-based approaches, the proposed methodology offers improved interpretability, reduced data requirements, and robust predictive capability. These results demonstrate the potential of combining computer vision and ML for data-driven quality inspection and process optimization in laser manufacturing. Furthermore, the framework can be extended to a wide range of laser-material processing applications requiring automated defect quantification and explainable quality assessment.

## Figures and Tables

**Table 1:** Experimental conditions and fs laser processing parameters used for Al foil cutting.

Parameter	Value	Unit	Type
Pulse duration	200	fs	Fixed
Wavelength	1030	nm	Fixed
Repetition rate	1	MHz	Fixed
Scanning speed	6	m s^−1^	Fixed
Spot diameter	30	µm	Fixed
Laser power	5, 10, 20, 30	W	Variable
Laser fluence	0.7, 1.4, 2.8, 4.2	J cm^−2^	Variable
Number of scans	10, 20, 30, 40, 50, 60, 70	-	Variable

## Data Availability

The authors declare that the data supporting the findings of this study are available within the article.

## Abbreviations

The following abbreviations are used in this manuscript:

Al	Aluminum
HAZ	Heat-Affected Zone
fs	Femtosecond
ML	Machine Learning
XGBoost	eXtreme Gradient Boosting
CNN	Convolutional Neural Networks
SEM	Scanning Electron Microscopy
